# A randomised controlled trial is not a pilot trial simply because it uses a surrogate endpoint

**DOI:** 10.1186/s40814-018-0324-2

**Published:** 2018-07-30

**Authors:** M. J. Campbell, G. A. Lancaster, S. M. Eldridge

**Affiliations:** 1 ScHARR, University of Sheffield, S1 4DA, Sheffield, UK; 2 Research Institute for Primary Care and Health Sciences, Keele University, Keele, UK; 3 Centre for Primary Care and Public Health, Queen Mary University of London, London, UK

**Keywords:** Pilot trials, Surrogate endpoints, Randomised controlled trials, CONSORT

## Abstract

**Background:**

It has been argued that true endpoints (or ‘hard’ endpoints) for clinical trials, which are meaningful to clinicians, researchers and patients alike, are limited to those that measure health status, survival and cost. Other endpoints are termed 'surrogate' endpoints and are intended to substitute and predict the true endpoint.  A number of trials that describe using surrogate endpoints use the term ‘pilot’ in the title of the paper but the reason for this, as related by the authors, is the use of these surrogate endpoints in the trial. The conduct and reporting of such a trial may follow the traditional pattern for a conventional randomised controlled trial (RCT) as defined by the original CONSORT statement, with power-based sample size calculations, and significance tests of the results. However, this is contrary to the guidelines of the CONSORT extension for the reporting of pilot trials.

**Main body:**

We review the definition of a surrogate endpoint and the use of surrogate endpoints in clinical trials. We consider to what extent a trial could be considered a pilot trial if it uses a surrogate endpoint and discuss two examples that illustrate current practice.

**Conclusion:**

Trials which use surrogate endpoints should only be described as ‘pilot’ when a definitive trial is a distinct possibility and the authors consider conditions which would indicate whether the definitive main trial was worthwhile and feasible. Simply because a trial uses a surrogate endpoint is not justification for calling it a pilot trial.

## Background

The recent discussion on pilot studies has come up with a clear picture of what a pilot trial should be and how it should be conducted and reported [1–3]. Here, the focus should be on preparation for a future definitive trial with feasibility objectives that address uncertainties in the study design. However, trials that use surrogate endpoints have also been described as ‘pilot trials’, and yet these may not differ from a main trial testing effectiveness in any other way, other than the endpoint used. This paper discusses to what extent the use of surrogate endpoints can justify the description of a trial as a pilot trial.

## Surrogate endpoints

Weintraub et al. [4] have argued that the true endpoints for randomised controlled trials (RCTs), those which are meaningful to clinicians, researchers and patients alike, are limited to health status, survival and cost. Sometimes, these are termed ‘hard’ endpoints. All other measures may then be seen as what are known as ‘surrogate’ endpoints. They also have argued that even serious events such as myocardial infarction and stroke may be considered surrogates, as their effect is to adversely affect the critical endpoints of health status, survival, and cost. Contentiously, this would mean that most clinical trials are run with surrogate endpoints. However, the reason for choosing a surrogate endpoint is that often it is difficult to run trials with true endpoints. Thus, surrogates have to be variables that are good predictors of the true endpoints. For example, high blood pressure is usually symptom-less and so does not affect health status, but it is highly correlated with events such as strokes and death and is a commonly used surrogate [4].

A surrogate is a relatively easy-to-measure endpoint which is available over a reasonably short time-frame that is used in place of the true endpoint. Surrogates are ‘Biomarker or intermediate end point intended to substitute and predict for patient-relevant final end point’ [5]*.* Surrogates are usually continuous variables which will allow for much smaller sample sizes than dichotomous variables as well as shorter periods of follow-up and lower costs. Thus, compared with clinical endpoint trials, studies with surrogate endpoints can be conducted rapidly and with much less resource use and expense than true endpoint studies.

Recent studies have challenged the assumption that reliance on surrogates can accurately predict the effect of treatment on clinical or true outcomes. The classic example is a study of type-I anti-arrhythmic drugs in patients who had heart rhythm disturbances after myocardial infarction. Among these patients, a trial showed that the anti-arrhythmic drugs encainide and flecainide decreased electrocardiographic (ECG) instances of arrhythmia, which was the surrogate end point. For this reason, these drugs were used regularly for this type of patient. When tested in an RCT with hard endpoints, however, patients who took encainide and flecainide turned out to be more than twice as likely to die from cardiac arrest or other causes than those randomised to placebo [6]. In effect, the drugs reduced arrhythmia but killed people. Other examples include oral hypoglycaemic drugs that reduce HbA1c but increase the risk of cardiovascular events [7]; antihypertensive drugs that do not reduce the risk of stroke [8]; and drugs that improve cholesterol profiles but do not reduce cardiovascular events [9]. Kemp and Prasad [10] showed that between 2004 and 2008, 36 oncology drugs were approved on the basis of a surrogate endpoint (such as progression free survival). With a median follow-up of 4.4 years, only 5 (14%) had been shown to give an improvement in overall survival (a ‘hard’ endpoint) in a randomised controlled trial.

Pilot trials are trials done before a main trial, designed to support the development of a future definitive RCT [1]. ‘Definitive’ in this context means an appropriately powered study focusing on effectiveness or efficacy. A definitive trial could still use a surrogate endpoint, provided that endpoint has been rigorously assessed as being an effective substitute for a hard endpoint. However, there is still no clear consensus. The question is whether trials done using surrogate endpoints can be considered ‘pilots’? Two relevant points in the recent CONSORT extension to pilot trials are that ‘the number of participants in a pilot study should be based on the feasibility objectives’ and ‘formal hypothesis testing for effectiveness (or efficacy) is not recommended’ [2, 3]. The aim of a pilot trial should be not to assess effectiveness (or efficacy) of a treatment, but rather to decide whether a larger definitive trial is worthwhile and feasible [2, 3].

## Examples

An example of a trial that is described as a pilot trial and uses a surrogate endpoint is that of Krarup et al. [11]. They describe the ExSTroke Pilot trial, to examine the benefits of exercise in patients who have had a stroke. They intended to recruit 300 subjects, but this was powered on a postulated difference in treatment groups from a surrogate endpoint, the Physical Activity Scale for the Elderly (PACE). The reason for the term ‘pilot’ in the title could be inferred because the study was not powered for recurrent stroke, myocardial infarction, or mortality, which may be regarded as hard endpoints. The results were published as a randomised controlled trial using conventional tests of significance although the word ‘pilot’ was retained in the title [12]. The authors admit that the study was not powered to show an effect of physical training on recurrent stroke, acute myocardial infarction, or survival, which would have needed more than ten times the sample size. However, to be consistent with the CONSORT extension, the trial should only deserve the label ‘pilot’ if there were clear criteria to decide on whether to conduct a subsequent trial using clinically meaningful outcomes, and a clear intention of conducting such a trial if the criteria were met. Otherwise, the title should simple state that the study is a randomised controlled trial that uses surrogate end points. Thus, if the ExStroke trial was to be regarded as a pilot, it could have specified what size difference in the PACE outcome would have justified further follow up for stroke and death.

The DECADE trial protocol gives another example of the use of surrogate endpoint in a pilot trial [13]. In this case one of the endpoints is the score for the 10-year risk of cardiovascular disease. However, here the authors do not power the study on a change in the risk score and rather state their objective is to find out whether the DECADE intervention is promising and whether a larger multi-centre randomised controlled trial is feasible. Their objectives were: to test DECADE regarding its usability and acceptance in primary care; to test the feasibility of the randomised study design; to generate initial data on the potential effects of DECADE in terms of patient knowledge, skills, confidence and behaviour critical for coping with a chronic illness, behavioural changes and clinical outcomes. This study thus falls within the scope of the definition of a pilot trial [1].

## Discussion

How a study is described, particularly in a title, is important because it influences how the paper describing the study is retrieved, and also suggests how the study should be analysed and reported. Thus, a trial which is described as a ‘pilot’ trial leads to the expectation that it is preparatory to a definitive trial, and the main reporting would be to enable the reader to decide whether a definitive trial was worthwhile and feasible. There is often an implicit assumption that a trial using a surrogate endpoint should be followed by a definitive trial, and so could be regarded as a pilot. However, if the surrogate has been previously rigorously assessed as a valid substitute for a hard endpoint, and the investigators are willing to discontinue or not recommend the new treatment based on the results of this trial, a further trial with hard endpoints is unnecessary. If, on the other hand, the surrogate endpoint has not been rigorously evaluated previously and part of the current study is devoted to choosing a suitable endpoint in a subsequent main trial, then the study may reasonably be described as a pilot. There is still much discussion about the best methods to validate surrogate endpoints [14]. We do not believe that studies to validate a surrogate endpoint should be described as pilot studies either. Such a study could be part of a large scale cohort study, and not related to any particular treatment.

A related issue is that of phase II, or ‘proof of concept’ drug trials. These form part of a drug development programme and may well be followed by a phase III drug trial if the sponsor thinks the results look promising. These may use surrogate endpoints but are not usually described as ‘pilot’ trials and so are not part of this commentary.

In reviewing the evidence for this commentary, we have found there are few surrogates that can be used with confidence as a substitute for a hard endpoint and it is rare for trials (except Phase II drug trials) which use surrogate endpoints to be followed by trials using definitive endpoints. Surrogate endpoints on average give larger effect sizes [15]. Many reported RCTS use surrogates and in fact more than 40% of pivotal trials used as the basis for approval of new indications used a surrogate as the primary endpoint [5]. As a general reporting issue, trials that use surrogate endpoints should clearly explain that the endpoints are surrogates, and if possible should report how these endpoints were validated so that they could be used with confidence as substitutes for hard endpoints.

## Conclusions

For the reasons outlined in this commentary, we believe that the use of the word ‘pilot’ to describe a study that uses a surrogate endpoint, but in all other respects is a conventional RCT, should be discouraged.
